# Self-parameterized active contours based on regional edge structure for medical image segmentation

**DOI:** 10.1186/2193-1801-3-424

**Published:** 2014-08-11

**Authors:** Eleftheria A Mylona, Michalis A Savelonas, Dimitris Maroulis

**Affiliations:** Department of Informatics and Telecommunications, National and Kapodistrian University of Athens, 15703 Panepistimiopolis, Athens Greece

**Keywords:** Active contours, Unsupervised parameterization, Structure tensors, Medical image segmentation

## Abstract

This work introduces a novel framework for unsupervised parameterization of region-based active contour regularization and data fidelity terms, which is applied for medical image segmentation. The work aims to relieve *MDs* from the laborious, time-consuming task of empirical parameterization and bolster the objectivity of the segmentation results. The proposed framework is inspired by an observed isomorphism between the eigenvalues of structure tensors and active contour parameters. Both may act as descriptors of the orientation coherence in regions containing edges. The experimental results demonstrate that the proposed framework maintains a high segmentation quality without the need of trial-and-error parameter adjustment.

## Introduction

Most medical image segmentation methods are parameterized empirically by medical doctors (*MDs*), who are unfamiliar with the intrinsic mechanisms of the underlying algorithms. Consequently, they depend on technical support by engineers. A key challenge is to develop reliable unsupervised parameterization approaches in order to set *MDs* free from the cumbersome and time-consuming process of parameter tuning, as well as to bolster the objectivity and reliability of the segmentation results.

Region-based active contours are prominent in medical image segmentation, due to their inherent noise-filtering mechanism (Ferrari et al. 2004; Liao et al. 2011; Petroudi et al. 2010; Xu et al. 2010). Data fidelity and regularization parameters are of critical importance in region-based active contour segmentation, since they act as amplifiers on the forces guiding contour evolution (Chan & Vese 2001; Li et al. 2010; Liu et al. 2011; Sundaramoorthi et al. 2008). Parameter values are typically adjusted in an empirical fashion and kept fixed for the entire spatial extent of an image. Thus, forces are uniformly weighted until convergence, regardless of the local image content.

Researchers have tried to confront the issue of empirical parameterization of region-based active contours by balancing the trade-off between regularization and data fidelity forces. However, each individual parameter is still empirically adjusted, as in the case of (Ma & Yu 2010). McIntosh and Hamarneh (McIntosh & Hamarneh 2007) adapt regularization weights across a set of images. Even though one weight may be optimal for some regions in an image, the same weight may not be optimal for all regions. Moreover, the data fidelity parameter is still empirically determined. Erdem and Tari (Erdem & Tari 2009) utilize data-driven local cues focusing on edge consistency and texture cues. Nevertheless, this method requires technical skills from the end-user. Dong et al. (2008) present an algorithm to capture brain aneurysms from the vascular tree, by varying the regularization term based on the surface curvature of a pre-segmented vessel. However, the regularization weight does not rely on image content. On the contrary, it depends on the shape of the target region, thus limiting the applicability of the method on different target shapes.

This work introduces a novel framework for unsupervised parameterization of region-based active contours, which is applicable on medical image segmentation. The proposed framework is inspired by the observation of an isomorphism between the eigenvalues of structure tensors and the active contour regularization and data fidelity parameters. The former are capable of describing the orientation coherence of regions containing edges. In a similar fashion, active contour parameters can be derived from a function encoding orientation coherence. The function used in the context of the proposed framework is the orientation entropy *(OE)*. This measure obtains low values in structured regions, which contain edges with low orientation variability, and high values in unstructured regions, which contain edges of multiple orientations. Accordingly, *OE* is capable to adjust forces driving the contour away from unstructured edge regions and guide it towards more structured ones, which are naturally associated with the boundaries of medical objects.

Regularization and data fidelity terms are self-parameterized in a spatially-varying fashion, as dictated by *OE*, facilitating the identification of the actual target boundaries, without the requirement for empirical fine-tuning or user-intervention. A byproduct of the proposed framework is that iterations dedicated to false local minima are bypassed, speeding up contour convergence. Any erroneous behavior in the early or middle stages of contour evolution is not propagated, since parameters hinge on information obtained solely from the image content and not from the possibly misleading contour shape.

It should be stressed that the proposed framework: 1) is not focused on convergence acceleration, which is only a byproduct, 2) is not proposed to substitute or outperform any of the numerous state-of-the-art active contour variations. Instead of these, the proposed framework primarily aims at unsupervised parameterization of region-based active contours, so as to achieve a segmentation quality which is comparable to the one obtained by the empirically fine-tuned version. In addition, the process for the identification of edge regions should not be confused with traditional edge detection, which focuses on single edges, often generated by noise.

The proposed framework is applicable to various medical imaging modalities and due to its simplicity and flexibility, can be embedded in various region-based active contour variations. Moreover, it is not sensitive on alterations in the settings of the acquisition devices. Such alterations, often required in clinical practice, naturally affect the acquired image features. As a result, in the case of empirical parameterization, manual fine-tuning might be required on a per-image basis, raising doubts on the actual value of a computational segmentation approach. A preliminary variation of this work appeared in (Mylona et al. 2012). Although the variation proposed in this study obtains high segmentation quality, it is not based on the valuable directional information derived by multi-directional filtering, whereas it has only been applied on a limited number of images.

The remainder of this paper is organized as follows: Section 2 describes the proposed framework and Section 3 presents the experimental results. The conclusions of this study are summarized in Section 4.

## Proposed framework

**Figure 1:**
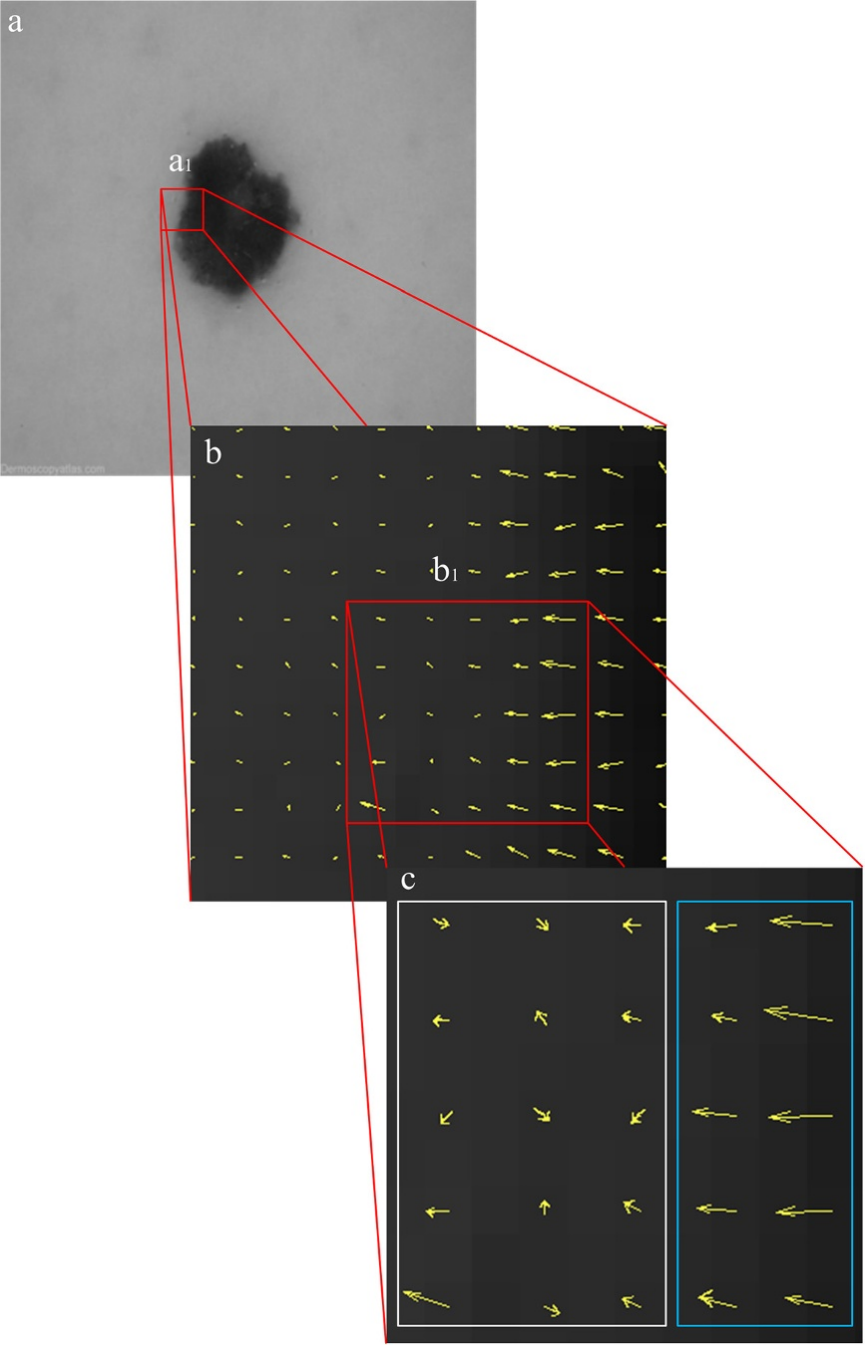
**Structure tensor field of target and non-target edge regions. a)** Dermoscopy image obtained by the Dermoscopy Atlas database containing a skin lesion, **b)** the structure tensor field and, **c)** the zoomed region b_1_, where white and blue rectangle corresponds to non-target and target edge region, respectively.

The proposed framework is tailored upon the structure tensor eigenvalues (Tschumperlé & Deriche 2005). Structure tensors are able to describe the orientation coherence in the proximity of edges. Providing that an image region contains edges of approximately constant orientation, or edges of multiple orientations, it can be identified by means of a structure tensor as a structured or unstructured edge region, respectively. The boundaries of medical objects are naturally associated with structured edge regions, whereas unstructured edge regions are associated with noise, artifacts and/or background clutter. In this light, structure tensors are capable of providing maps of target and non-target edge regions in the context of a medical imaging application. This is visualized with a real example in Figure 1, which illustrates: a) a dermoscopy image obtained by the Dermoscopy Atlas database (http://www.dermoscopyatlas.com) containing a skin lesion, where a_1_ corresponds to a region containing both non-target and target edge regions, b) the structure tensor field of the zoomed region a_1_ depicting orientations and, c) the zoomed region b_1_, where white and blue rectangle corresponds to the non-target and target edge region, respectively. It is evident that, multiple orientations appear in non-target, unstructured edge regions, whereas approximately constant orientation appears in target, structured edge regions.

In Weickert’s *diffusion* model (Weickert & Scharr 2002), a structure tensor *D* is capable of describing the orientation of an edge region and is defined as:
1

where *I* is the input image, *v*_1_, *v*_2_ are orthonormal eigenvectors and *λ*_1_, *λ*_2_ are the corresponding eigenvalues given by:
2

where the + sign belongs to *λ*_1_. It is worth noting that *λ*_1_ is the principal eigenvalue and is longitudinal with respect to the principal axis of the tensor ellipsoid, whereas *λ*_2_ is the minor eigenvalue and is vertical with respect to the same principal axis. Additionally, *λ*_1_ and *λ*_2_ integrate the variation of intensity values within a region and reflect the orientation coherence along the corresponding eigenvectors.

The isomorphism of the above formalism with region-based active contours can be identified if we consider the general form of the active contour energy functional:
3

where *E*_*reg*_ and *E*_*df*_ are associated with regularization and data fidelity energy terms, respectively, whereas *w*_*reg*_ and *w*_*df*_ are weighting parameters. Regularization forces are most likely tangent to the principal axis of the contour whereas data fidelity forces are vertical to this axis, attracting the contour towards target edges. It is tempting to notice that if we associate the principal axis of the tensor ellipsoid with the principal axis of the contour, the regularization weight *w*_*reg*_ corresponds to the same direction as the principal eigenvalue *λ*_1_, whereas the data fidelity weight *w*_*df*_ corresponds to the same direction as the minor eigenvalue *λ*_2_. This premise indicates a link between the active contour parameters and the eigenvalues of the structure tensor.

Inspired by this observation, regularization and data fidelity parameters are set to reflect the orientation coherence of edge regions in a similar fashion to Weickert’s diffusion model. This can be achieved by means of the orientation entropy (*OE)*. This measure can be calculated as follows:
45

where *I*_*jk*_ is a subband image generated by a multi-directional filtering method (Do & Vetterli 2005), *OE*_*jk*_ is the *OE* of the subband image *I*_*jk*_ in the *k*^*th*^ direction and the *j*^*th*^ level of decomposition, *p*_*jk*_ is the probability function, *M*_*jk*_ is the row size and *N*_*jk*_ the column size of the subband image. It should be highlighted that *OE* is calculated on each directional frequency of the original image, generated by the contourlet transform (*CT*) (Do & Vetterli 2005) and not on the original image, since the former is capable to highlight the orientation coherence in edge regions.

Regularization and data fidelity parameters are matrices of the same dimensions as the original image and can be calculated according to the following equations:
6

The main idea is to navigate the contour towards structured, target regions (blue rectangle of Figure 1(c)) in the early stages of evolution. This is achieved by assigning high values of *OE* to *w*_*df*_ which will appropriately amplify data fidelity forces in randomly oriented, high-entropy regions. Hence, iterations dedicated to erroneous local minima will be bypassed, speeding up contour convergence towards target edges. It should be noted that both parameters are calculated only once at initialization. The aim is to guide the contour directly to target edge regions, already from the beginning, and to prevent any erroneous behavior during evolution by ‘constantly reminding’ where the target edge regions lie. Furthermore, by setting regularization terms as the reciprocal of data fidelity terms, the proposed framework achieves a balanced trade-off between regularization and data fidelity parameters. This is of primary importance in cases of unstructured edge regions where data provide a less reliable clue than contour regularization.

**Figure 2:**
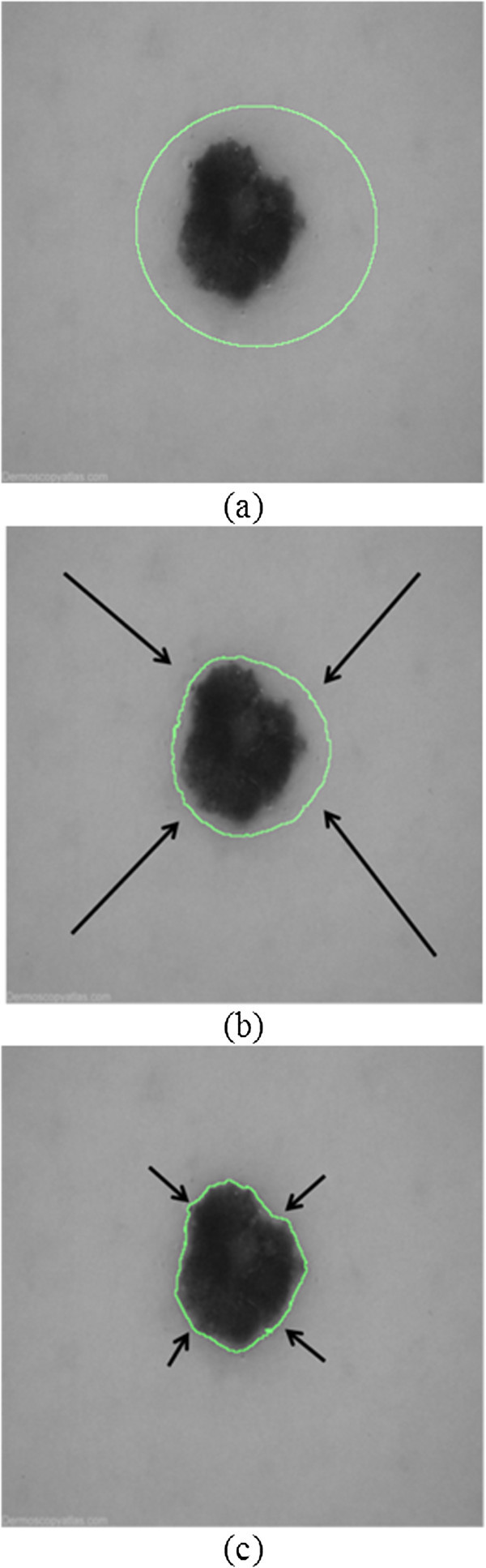
**Contour evolution of the proposed framework** **.** **a)** contour initialization on the dermoscopy image of Figure 1, **b)** early stage of contour evolution, **c)** later stage of contour evolution (black arrows represent region-based forces).

Figure 2 demonstrates the contour evolution achieved by the proposed framework. Figure 2(a) depicts the contour initialized as a green circle on the dermoscopy image of Figure 1. Figure 2(b) depicts an early stage of contour evolution, whereas Figure 2(c) depicts a later stage. For as long as the contour lies in unstructured edge regions associated with background clutter, *OE* obtains high values and the data fidelity parameter is increased. Thus, region-based forces (long black arrows) are appropriately amplified; repelling the contour away from such regions and guiding it towards more structured ones (Figure 2(b)). Once the contour approximates the vicinity of structured edge regions, *OE* obtains low values and the data fidelity parameter is decreased. Hence, region-based forces (short black arrows) are appropriately reduced in order to facilitate convergence (Figure 2(c)).

## Results

The proposed framework is embedded into the Chan-Vese model (Chan & Vese 2001) by replacing the empirically fixed parameters with the automatically adjusted ones. Rather than comparing one active contour method with another, the experiments to follow aim to evaluate the effectiveness of the proposed framework by comparing the segmentation performance of the unsupervised versus the empirically fine-tuned version. The Chan-Vese model determines the level set evolution by solving the following equation:
7

where *ϕ* is the level set function, *I* the observed image, *c*_1_, *c*_2_ the average intensities inside and outside of the contour, respectively,  the fixed regularization parameter and  the fixed data fidelity parameter. For the empirical case, parameters  and  obtain the optimal values 0.006 ⋅ 255^2^ and 1, respectively, according to the original paper (Chan & Vese 2001). For the proposed framework, the regularization and data fidelity parameters are automatically calculated according to (6). In addition, contour is initialized in the proximity of the target region and remains the same for both the proposed framework and the empirically fine-tuned version so as to facilitate fair comparisons.

Experiments are conducted on databases of various medical imaging modalities so as to confirm the framework’s generality with respect to image content. All image modalities were investigated by *MDs* who provided ground truth images. The shape of all abnormalities as well as the irregularity of their margins are malignant risk factors which are highly considered by *MDs* before proceeding to fine needle aspiration biopsy.

The first database consists of 50 mammographic images containing abnormalities randomly obtained by the mini-MIAS database (Suckling et al. 1994). The background tissue is characterized as: a) fatty, b) fatty-glandular and c) dense-glandular, whereas the abnormality is classified as: a) well-defined/circumscribed and b) ill-defined. In terms of its severity, the abnormality is defined as benign or malignant.

The second database consists of 45 thyroid ultrasound images containing hypoechoic nodules provided by the Radiology Department of Euromedica S.A., Greece. All ultrasound images were acquired using a digital ultrasound imaging system HDI 3000 ATL with a 5-12 MHz linear transducer. Instrument settings were fixed accordingly to the built-in ‘SmallPartTest’ Philips protocol, magnification was set to 1:1 and dynamic range was set to 150 dB/C4. Hypoechoic nodules with regular boundaries may represent follicular neoplasms of medium-risk, whereas hypoechoic nodules with irregular boundaries are considered suspicious for malignancy and may represent thyroid carcinomas.

The third database consists of 32 endoscopy frame images containing polyps provided by the Gastroenterology Section, Department of Pathophysiology, Medical School, University of Athens, Greece and partially by the Section for Minimal Invasive Surgery, University of Tübingen, Germany. The endoscopic data was acquired from sixty-six different patients with an Olympus CF-100 HL endoscope. All frame images consist of small size adenomatous polyps which are not easily detectable and are more likely to become malignant.

The fourth database consists of 40 labial teeth and gingiva photographic images randomly obtained by the LTG-IDB database (Eckhard et al. 2012) created by the Color Imaging Lab at the Optics Department of the University of Granada, Spain. For the image acquisition, a Canon EOS 7D digital single-lens reflex color camera combined with a Canon EFS 18-135 mm standard zoom lens was used. The scope of this database usage is the task of teeth/non-teeth segmentation.

**Figure 3:**
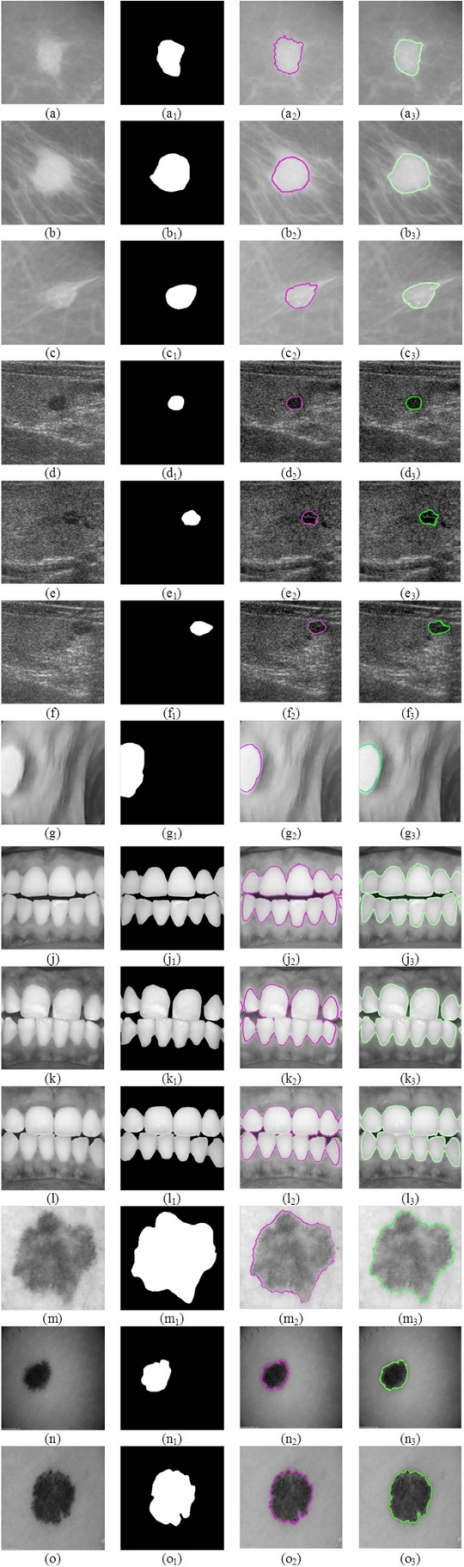
**Segmentation results obtained by the unsupervised version and the empirically fine-tuned version. (a)-(c)** Mammographic images containing abnormalities, **(d)-(f)** thyroid ultrasound images containing nodules, **(g)-(i)** endoscopy images containing polyps, **(j)-(l)** labial teeth and gingiva photographic images, **(m)-(o)** dermoscopy images containing skin lesions, **(a**_**1**_ **)-(o**_**1**_ **)** corresponding ground truth images, **(a**_**2**_ **)-(o**_**2**_ **)** segmentation results of the empirically fine-tuned version, **(a**_**3**_ **)-(o**_**3**_ **)** segmentation results of the unsupervised version.

The fifth database consists of 50 dermoscopy images containing skin lesions randomly obtained by the Dermoscopy Atlas, which is provided by the Skin Cancer College of Australia and New Zealand. The more asymmetrical the distribution of the pigmented lesion is, the more it is considered as an evolving growing nevus or a melanoma. Figure 3 illustrates segmentation results obtained by the unsupervised version using the proposed framework, as well as by the empirically fine-tuned version.

It should be noted that the size of the images obtained by the first and second database is 256 × 256 and the size of the images obtained by the third, fourth and fifth databases is 320 × 320. For an image of size 256 × 256, an image grid of size 32 × 32 is considered suitable and is fed into *CT* through an iterative procedure. The image grid is further decomposed to four subbands of size 16 × 16. For an image of size 320 × 320, an image grid of size 40 × 40 is considered suitable for *CT* decomposition. In this case, the obtained four subbands’ size is 20 × 20. The size of the image grid is experimentally determined as the minimum of the negative power of two of the original image size, which maintains an edge region.

The segmentation results presented in Figure 3 demonstrate that the proposed framework achieves *comparable segmentation quality* to the one obtained by the empirically fine-tuned version *in an unsupervised fashion*.

The experimental results are quantitatively evaluated by means of two metrics:

- the Tannimoto coefficient (*TC*) (Crum et al. 2006), which is defined by: 8

where *A* is the region identified by the segmentation method under evaluation, *B* is the ground truth region and *N*() indicates the number of pixels of the enclosed region and

b) the Hausdorff distance *H* (Huttenlocher et al. 1993) defined as: 9

where  is called the directed Hausdorff distance from *A* to *B*, *A* is the ground truth set, *B* the set under evaluation and *a, b* the points defined in sets *A*, *B*, respectively.

The unsupervised version achieves an average *TC* and *H* value of 82.9 ± 1.6% and 42.3 ± 1.3 mm, respectively with regards to all images tested, which is comparable to the *TC* and *H* value of 80.7 ± 1.8% and 40.9 ± 1.5 mm, respectively obtained by the empirically fine-tuned version. This comparable segmentation accuracy verifies the value of the proposed framework for unsupervised parameter adjustment.

**Figure 4:**
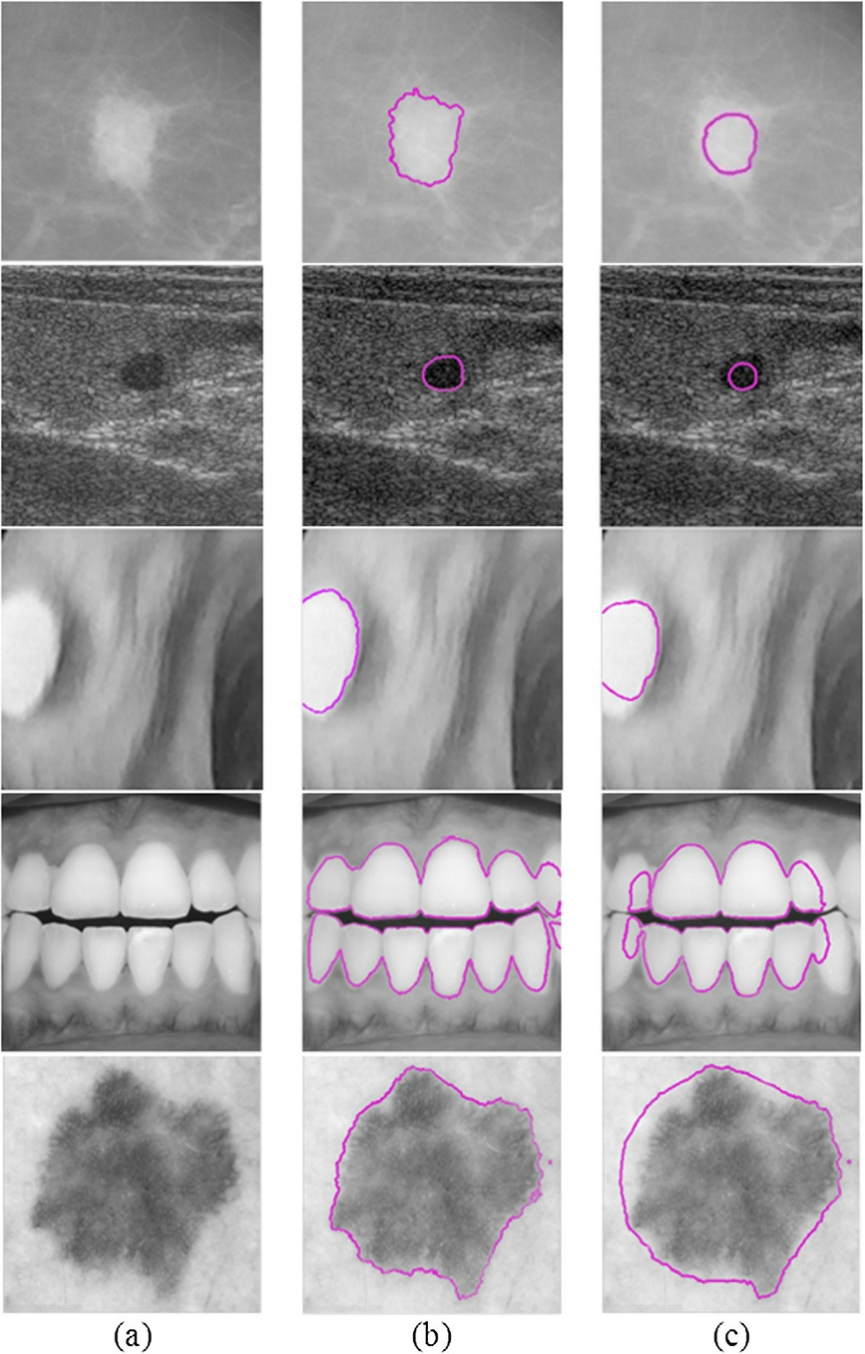
**Segmentation results of the empirically fine-tuned version and the randomly-tuned version. a)** Sample images of each utilized database, **b)** segmentation results obtained by the empirically fine-tuned version and, **c)** segmentation results obtained by the randomly-tuned version.

Aiming at highlighting the importance of the proposed framework, further experiments are conducted in order to investigate the sensitivity of the empirical version to small alterations of parameters. The empirical version, except of being adjusted with optimal parameters, is also adjusted with parameters which are randomly set. Parameters  and  were set to randomly selected values, which fluctuated up to 10% from the optimal ones. Figure 4 depicts: a) sample images of each utilized database, b) segmentation results obtained by the empirically fine-tuned version and, c) segmentation results obtained by one case of the randomly-tuned version where  and  were randomly set to 0.001 · 255^2^ and 0.1, respectively. It is evident that the segmentation results obtained by the randomly-tuned version differ significantly from the ones obtained by the fine-tuned version. The randomly-tuned version achieves average *TC* and *H* of 58.3 ± 1.7% and 31.2 ± 1.9 mm, respectively, with respect to all values tested, which differ significantly from the ones achieved by the fine-tuned version. Hence, the empirical version is sensitive even to small alterations of parameters and the segmentation results are highly questioned. On the contrary, the proposed framework achieves a high segmentation quality in an unsupervised fashion, endowing segmentation results with objectivity.

**Figure 5:**
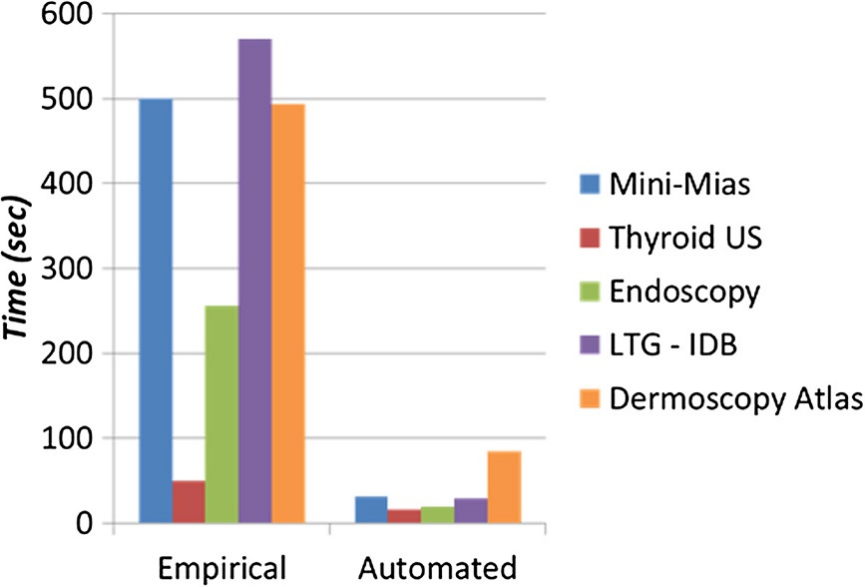
**Elapsed time of contour convergence for both empirical and unsupervised parameterizations for each utilized database.**

Figure 5 illustrates the elapsed time of contour convergence for both empirical and unsupervised versions, for each reference database. It is evident that the unsupervised version converges approximately 5-10 times faster than the empirical version. This is because region-based forces guiding contour evolution are appropriately amplified in randomly oriented, high entropy regions, driving the contour away (Figure 2). Hence, iterations dedicated to false local minima, which are associated with such regions, are avoided. On the contrary, in the case of empirical parameterization, region-based forces are uniformly weighted irrespectively of *OE*. Hence, the contour is delayed in false local minima and kept away from target edge regions for more iterations. It should be stressed that the convergence acceleration is a byproduct of the proposed framework and not its main motivation, which is the capability of contour self-parameterization.

## Discussion

In this work, a novel framework for unsupervised adjustment of active contour regularization and data fidelity parameters is presented and applied for medical image segmentation. The work is motivated by the need for unsupervised parameterization, which is prominent in medical imaging applications, so as to relieve MDs from the laborious, time-consuming task of empirical parameterization and bolster the objectivity of the segmentation results.

The proposed framework is inspired by an isomorphism between these parameters and the eigenvalues of structure tensors. Considering that the latter provide essential information associated with the orientation coherence of edge regions, we encode this information in parameters by means of *OE*, which obtains high values in unstructured edge regions and low values in structured ones. In this way, region-based forces drive the contour away from non-target, unstructured regions and navigate it towards the target, structured ones, which are naturally associated with the boundaries of medical objects (Figure 1).

The proposed framework is validated on several medical image databases by comparing its segmentation performance with the empirically fine-tuned version. The experimental results demonstrate that the unsupervised version maintains a high segmentation quality, comparable to the one obtained empirically, yet in an unsupervised fashion. Hence, *MDs* are set free from the laborious process of empirical parameterization and the objectivity of the results is enhanced. Moreover, contour convergence is accelerated.

Although this work addresses the medical imaging domain, demonstrating the effectiveness of the proposed framework in various medical imaging modalities, in principle it can also be applied to other imaging domains. This could be the subject of a future study, since it is out of the scope of this work. Another future direction involves the integration of the proposed framework on different region-based active contour variations.
